# Allo-Priming Reverses Immunosenescence and May Restore Broad Respiratory Viral Protection and Vaccine Responsiveness to the Elderly: Results of a Phase I/II Clinical Trial

**DOI:** 10.3390/vaccines13050463

**Published:** 2025-04-25

**Authors:** Canhui Liu, Xiaochuan Yang, Jorge Paoli-Bruno, David Sikes, Alejandra V. Marin-Ruiz, Nicole Thomas, Ryan Shane, Michael Har-Noy

**Affiliations:** 1 Mirror Biologics, Inc., Wesley Chapel, FL 33544, USA; 2 Coral Research Clinical Corp, Miami, FL 33186, USA; 3 Florida Medical Clinic Orlando Health, Zephyrhills, FL 33542, USA; 4 Model Research, Tampa, FL 33615, USA; 5 Delray Physician Care Center, Delray Beach, FL 33445, USA

**Keywords:** Th1/Th2 balance, immunosenescence, heterologous immunity, cellular immunity, respiratory viral infection, interferon gamma, community-acquired pneumonia, cytopathic effect assay, clinical trial, elderly, trained immunity

## Abstract

Respiratory viral infections pose a significant health problem that disproportionately affects the elderly. With the aging worldwide population being less responsive to protective vaccines, there is an urgent need for strategies that can protect the elderly from community-acquired viral infections. Background/Objectives: Allo-priming is a novel immunomodulatory vaccine concept using allogeneic, living, activated Th1 cells that are rejected by the host, creating anti-alloantigen Th1 immunity, increasing Th1 titers. Th1 cells orchestrate cellular immunity, and the age-related decline in Th1 cells contributes to weakened cellular immune response in the elderly, which correlates with poor responsiveness to vaccines and increased susceptibility to respiratory viral infections. Increased Th1 cell titers in the elderly were hypothesized to reverse immunosenescence and restore cellular immune function. Restoration of cellular immune function was predicted to restore broad respiratory viral protection through a heterologous immune mechanism. Methods: A phase I/II, multi-center, open-label clinical trial was conducted in 40 healthy adults over 65 years of age to investigate the safety of allo-priming and the effects this vaccination strategy has on cellular immune function over time. Results: Allo-priming had a benign safety profile and significantly increased the titers of circulating Th1 cells. The increase in Th1 cells was shown to provide broad, self-amplifying respiratory viral protection over time in an ex vivo cytopathic effect assay without additional vaccinations and without any viral antigens included in the formulation, as well acting to increase neutralizing antibody titers in low-responding individuals previously vaccinated for COVID-19. Conclusions: These results provide support for an expanded clinical evaluation of this immunomodulatory vaccination strategy as a possible method to restore cellular immune competence to the elderly and provide broad heterologous immune protection from respiratory viral infections without the need for frequent vaccine re-formulations or booster shots (National Library of Medicine: NCT04441047).

## 1. Introduction

Respiratory viral infections have a disproportionate impact on the elderly population. Upon infection, healthy young adults generally only experience mild to moderate upper respiratory tract illness, while the elderly, particularly those with co-morbidities, more frequently experience severe bronchiolitis and pneumonia with high morbidity and mortality [1,2,3]. Vaccination can be a protective strategy against respiratory viruses; however, most current vaccines are less effective in the elderly [4,5]. Therefore, a growing medical challenge exists to develop new vaccination strategies specifically designed to protect the elderly [6].

The current vaccination formulations generally aim to induce high titers of neutralizing antibody responses for protection. However, many respiratory viruses mutate rapidly, requiring frequent re-formulation in order to address new surface components that escape previously induced neutralizing antibodies. Moreover, respiratory viral particles often target entry into the epithelial cells lining the respiratory tract. Once inside of these cells, viruses can spread cell-to-cell, avoiding neutralizing antibodies in the mucus [7], suggesting that a cellular immune response may also be required for adequate viral control.

While cellular immunity is known to be vital for identifying and eliminating virus-infected cells [8], most respiratory viral vaccines currently in use were developed without focus on the cellular immune response [9,10]. Once viral infections reach the lower respiratory tract, the lack of cilia and mucus in this compartment leaves acute control of viral spread up to resident macrophages. Phagocytosis of infected cells by macrophages and subsequent antigen processing can result in viral-specific adaptive T-cell responses, leading to “sterilizing immunity” [11]. However, aging impairs the ability of alveolar macrophages to rapidly clear lower respiratory viral infections [12], thus making the elderly more susceptible to severe disease. Therefore, restoration of cellular immune competence in the elderly may be essential for protection from severe viral infections.

Age-related dysfunction of cellular immunity has been shown to contribute to the vulnerability of elderly individuals to viral infection [13,14], including progressive declines in both innate and adaptive cellular immune function [15,16]. Accordingly, viral vaccines that elicit cellular immune responses have become a highly desirable goal for vaccination protocols that target the elderly [17].

The decline in cellular immune function in the elderly is a condition referred to as “immunosenescence” [18,19]. Immunosenescence is characterized by suppressed adaptive immunity and dysfunctional innate immune responses to viral infection [20]. Reversal of immunosenescence and restoration of cellular immune function in the elderly is therefore a strategy that could enable the elderly immune system to respond to respiratory viral infections in a manner that resembles the immune responses observed in younger adults.

Interferons (IFNs) are key orchestrators of cellular immune responses, inhibiting viral entry, blocking viral replication and preventing the release of new viral particles [21]. Type I IFNs play a central role in anti-viral immunity [22]. However, type I IFN signaling and production is defective in the elderly [23]. IFN-γ, a type II IFN, is also a key cytokine involved in early protection during acute viral infection [24]. The presence of IFN-γ acts on both the infected cell and neighboring cells by creating an “anti-viral state”, a condition in which cells become resistant to viral replication [25]. Since Th1 cells are a major source of IFN-γ [26], our aim was to substitute for defective type I IFN signaling in the elderly by increasing Th1 cell titers and the release of IFN-γ.

When exposed to a pathogen for the first time, naïve lymphocytes are activated and differentiate to either Th1 or Th2; however, the pool of naïve lymphocytes with capacity to differentiate declines with age [27], limiting the diversity and depth of cellular immune responses to viruses. To overcome this limitation, we proposed vaccination with living allogeneic immune cells. Alloantigens are potent immunogens, as even the most severely suppressed immune systems are able to reject allogeneic grafts [28].

We developed a living, activated, non-genetically altered, allogeneic Th1 immune cell (AlloStim^®^) derived from healthy, intentionally mis-matched blood donors that will elicit an anti-alloantigen Th1 immune response upon rejection by the host. We have previously shown in a mouse model (Balb/c), which responds to antigens with a genetic Th2 bias [29], that rejection of mis-matched AlloStim^®^ could significantly increase Th1 cell titers despite the genetic Th2 bias [30]. We then proposed that repeated intradermal (ID) injections of AlloStim^®^ to the elderly, a strategy we called “allo-priming”, might increase the titers of circulating Th1 cells and provide the same broad immune protection against respiratory viruses, both known and unknown, in the elderly as occurs in the young [31]. This phase I/II clinical trial was designed to determine a clinical proof of concept for this hypothesis.

## 2. Materials and Methods

### 2.1. Ethical Approvals

This phase I/II, open-label clinical study was conducted at 4 outpatient clinics in the United States in accordance with Good Clinical Practice (GCP) guidelines. The study protocol was cleared by the US Food and Drug Administration (FDA) under an active Investigational New Drug (IND) application. The study documents were independently reviewed and approved by a central institutional review board (IRB). An independent data safety monitoring board (DSMB) reviewed clinical data for any safety signals. All participants provided written informed consent.

### 2.2. AlloStim^®^ Formulation

AlloStim^®^ is derived from healthy blood donors and has an identity phenotype of IFN-γ+, CD4+, CD45RO+, CD62L^lo^ and CD40L^hi^. AlloStim^®^ is formulated with microbeads that have anti-CD3/anti-CD28 monoclonal antibodies attached. The AlloStim^®^ cells, with beads attached at a 2:1 bead:cell ratio, were suspended at 1 × 10^7^ cells/mL in 0.5 mL of the formulation buffer containing PlasmaLyteA^®^ with 5% human serum albumin (HSA) and 2% dimethyl sulfoxide (DMSO). The formulated AlloStim^®^ cells were aliquoted into vials and cryopreserved in liquid nitrogen (LN2). The vials were transported to clinical sites in LN2 dry shipper containers. Prior to use, the vials were thawed and injected intradermally (ID) per protocol.

### 2.3. Study Participants

This study enrolled 40 healthy adults, 20 of whom were aged 65–74 years (mean: 70.6 yo; range: 65.1–74.6 yo) and 20 of whom were aged 75 years and older (mean: 79.7 yo; range: 76.6–89.9 yo). Eligible volunteers were screened to be in general good health via a physical exam and with complete blood counts, liver function, kidney function and inflammation marker clinical laboratory tests within acceptable normal reference ranges. Volunteers were excluded if they had a history of autoimmune disease; were HIV-positive; had a history of organ transplant or tissue allograft; or had any uncontrolled concurrent serious medical or psychiatric illness. SARS-CoV-2 (COVID-19) vaccination was required prior to enrollment, and influenza vaccination was allowed 28 days prior to the first dose and 28 days after the last dose. Influenza A (IAV) IgG (EIA) and influenza B (IBV) IgG (EIA) were determined at baseline and at day 336. Volunteers were screened for COVID-19 antigen over the duration of the study if they reported flu-like symptoms and at regular office visits.

### 2.4. Protocol

Volunteers were administered 0.5 mL of AlloStim^®^ via ID injection on day 0, day 3 or 4, day 7, day 10 or 11 and day 14. Peripheral blood samples (20 mL) were collected on days 0, 10, 28, 168 and 336 in EDTA lavender top tubes. Peripheral blood mononuclear cells (PBMCs) were isolated from peripheral blood via Ficoll-Hypaque density gradient centrifugation, washed and suspended at 1 × 10^7^ cells/mL in 90% fetal bovine serum (FBS) and 10% DMSO and stored in LN2 until evaluated for cellular immune function. Adverse events (AEs) were monitored in each volunteer at each dosing and during the 336-day evaluation period.

### 2.5. Mechanism of Action

**Figure 1:**
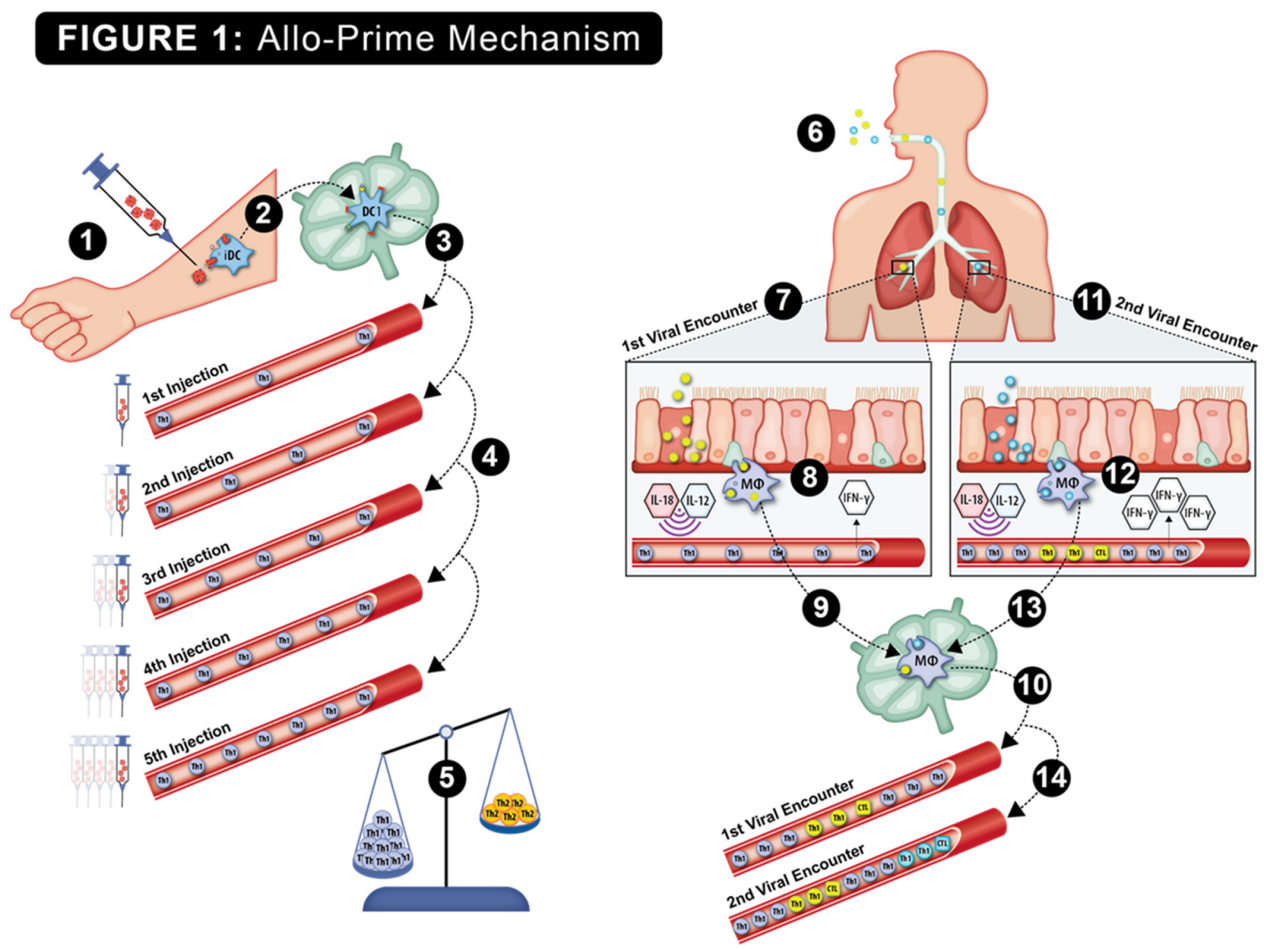
The putative step-by-step mechanism of action of allo-priming is shown: 1. Intradermal injection of AlloStim^®^. 2. The intentionally mis-matched allogeneic living AlloStim^®^ cells are rejected by the host immune system, and local immature DC (iDC) engulf and process the alloantigens in the context of Th1-steering danger signals released upon rejection of the allogeneic cells. The iDC mature to IL-12+ DC1, which express MHCI/II and CD80/86 co-stimulatory molecules, migrate to the draining lymph node and present alloantigens to cognate T cells. 3. Allo-specific Th1 cells enter the peripheral blood (shown in blue). 4. Upon each intradermal injection of AlloStim^®^, the titer of allo-specific Th1 cells in circulation increases. 5. The increases in circulating Th1 cells increase the Th1/Th2 balance. 6. Exposure to respiratory viruses in the environment initiates cellular immune responses. 7. Upon the first viral encounter (shown as a yellow virus), alveolar macrophages engulf viral-infected cells in the respiratory tract, which activates internal TLR7/8 and causes the release of cytokines such as IL-18 and IL-12, which act to activate bystander Th1 cells causing their release of IFN-γ in the microenvironment, creating an anti-viral state. 9. The alveolar macrophages that have engulfed viral-infected cells traffic to the draining lymph node and activate cognate viral-specific Th1/CTL. 10. Viral-specific Th1/CTL (shown as yellow cells) enter circulation, further increasing the Th1/Th2 balance. 11. Upon a second viral encounter (shown as a blue virus), alveolar macrophages engulf and process viral-infected cells and again cause bystander activation of Th1 cells, with increased release of IFN-γ. 13. The alveolar macrophages traffic to the draining lymph node and activate cognate Th1/CTL specific to the second virus. 14. These new viral-specific Th1/CTL (shown as light blue cells) enter circulation, further increasing the Th1/Th2 balance. In this manner, a self-amplifying increase in cellular immune function mediated by the modulated Th1/Th2 balance results upon each environmental encounter with respiratory viruses.

The hypothesized viral protection mechanism of allo-priming is shown in Figure 1. Host rejection of multiple AlloStim^®^ ID injections was predicted to elicit anti-alloantigen Th1 immunity, resulting in an increased titer of Th1 cells in circulation, which was predicted to correct the elderly Th1/Th2 imbalance. Upon exposure to a respiratory viral infection, viral entry into bronchial endothelial cells or engulfment of viral-infected cells by alveolar monocytes triggers internal Toll-like receptors (TLRs) and the release of inflammatory cytokines, such as IL-12 and IL-18. These cytokines are predicted to non-specifically activate the bystander allo-specific Th1 cells, causing their release of IFN-γ into the microenvironment, creating an “anti-viral state”. The creation of an anti-viral state would maintain a low viral burden, providing sufficient time for the development of an adaptive Th1/CTL viral-specific immune response that is capable of completely eliminating the offending virus (sterilizing immunity). After elimination of the first virus, the new viral-specific Th1/CTL cells now in circulation add to the existing allo-specific Th1 cells created after allo-priming, further increasing the Th1/Th2 balance. Later infection with a different virus was predicted to result in an amplified IFN-γ release through bystander activation of both the resident expanded allo-specific Th1 cells and the Th1/CTL cells specific to the first virus. Upon each subsequent viral encounter, bystander activation of memory allo-specific and viral-specific Th1 cells was predicted to cause increased release of IFN-γ and further expansion of the resident Th1 cells. In this manner, after remodeling the Th1/Th2 balance with allo-priming, subsequent environmental exposures to viruses were predicted to create a self-amplifying continuous strengthening of the cellular immune function over time. Thus, allo-priming would represent a type of trained immunity vaccine that could potentially provide broad protection from respiratory viral infections through viral cross-reactivity and through heterologous immunity (HI) mechanisms [32].

### 2.6. Cellular Immune Function Assays

PBMCs from longitudinal blood samples were analyzed for their ability to produce IFN-γ using the ELISPOT assay, and supernatants from activated PBMCs were analyzed for their ability to suppress viral lytic activity in a cytopathic effect (CPE) assay.

PBMCs isolated from day 0, 10, 28, 168 and 336 clinical research blood samples were incubated with cell activation cocktails: phorbol myristate acetate (PMA) (0.08 μM) with ionomycin (1.34 μM) for polyclonal T-cell activation; R848 (Resiquimod), a TRL7/8 agonist (1 µg/mL), to simulate viral infection with a single-stranded RNA (ssRNA) virus; and a lysate from AlloStim^®^ cells (250 µg/mL) to activate allo-specific T cells. The activation times were 24 h (PMA), 72 h (AlloStim^®^ lysate) and 120 h (R848), all evaluated in triplicate in 96-well plates.

For the CPE assay, the supernatants from the activated PBMCs were co-incubated with viral-infected cell line cultures, each cell line was selected for its sensitivity to lysis by the corresponding live virus: human respiratory syncytial virus (RSV) (ATCC, Cat# VR-26); human coronavirus 229E (ATCC, Cat# VR-740); influenza A virus (IAV) H1N1 (ATCC, Cat# VR-1894); and influenza B virus (IVB) Victoria Lineage strain B/Florida/78/2015 (ATCC, Cat# VR-1931). Human MRC-5 lung fibroblast cells (ATCC, Cat# CCL-171) were the target cells used for RSV and coronavirus 229E cultures, and human HEK-293 kidney epithelial cells (ATCC, Cat# CRL-1573) were the target cells used for IAV and IVB cultures.

The ELISPOT assay was performed using 96-well plates pre-coated with a monoclonal anti-human IFN-γ antibody, according to the manufacturer’s instructions (R&D Systems, Minneapolis, MN, USA, cat#EL285). PBMCs isolated from longitudinal clinical research blood samples were incubated at 1 × 10^6^ cells/mL in separate wells of a 96-well plate for each activation cocktail in triplicate. Secreted IFN-γ was captured on a polyvinylidene difluoride (PVDF) membrane, detected by another anti-human IFN-γ antibody, and visualized with alkaline phosphatase and 5-bromo-4-chloro-3-indolyl-phosphate/nitro blue tetrazolium (BCIP/NBT) and analyzed using a Cytation 7 image analyzer and BioTek Gen5 software (Agilent Technologies, Santa Clara, CA, USA).

**Figure 2:**
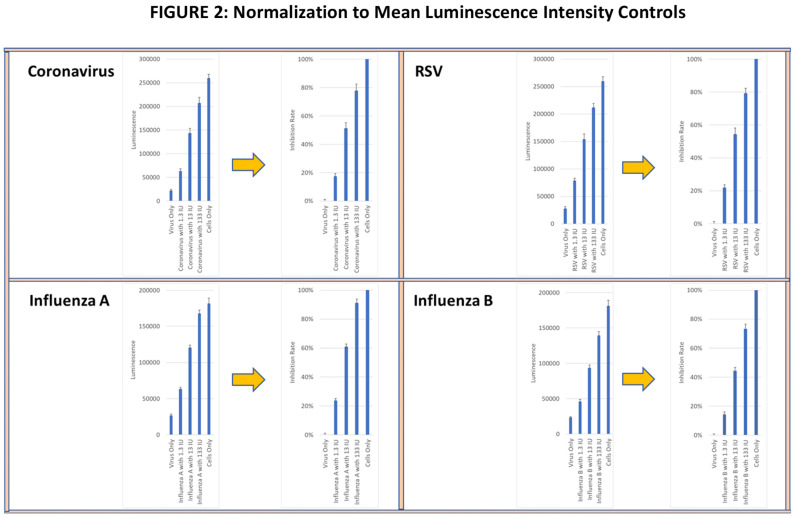
Viral lytic activity was determined in confluent cell cultures of MRC-5 lung fibroblasts incubated with live RSV and coronavirus and with HEK-293 kidney cells incubated with live influenza A and influenza B viruses. The mean ± SE of ATP luminescence intensity (LI) of viable cells was determined from triplicate wells of each virus culture is shown in the left panels. The LI of cultures with media alone for each virus was normalized to 100% viral suppression and the LI of cells cultured with virus alone was normalized to 0% viral suppression shown on the right panel. Recombinant IFN-γ at 1.3 IU, 13 IU and 133 IU was used as functional positive controls on the normalized panels.

In the CPE assay, anti-viral activity was measured based on the ability of supernatants from activated longitudinal PBMC samples to inhibit virus-induced cytopathology in infected cell lines. Cell lysis was determined using a cellular adenosine triphosphate (ATP) detection reagent (Real-Time-Glo^TM^ extracellular ATP assay, Promega, Madison, WI, USA), and luminescence intensity (LI) was measured using the Cytation 7 image analyzer. Viral infectivity and the corresponding tissue culture infective doses (TCIDs) were determined by serially diluting virus preparations and applying them to target cell lines for the specified exposure period. The virus dilution that produced a cytotoxic effect of >80% of the cultured cells (TCID80) was calculated and used for all subsequent studies. Wells with (virus alone) and without virus (media alone) were used as controls. The percent inhibition of the lytic activity of each virus was normalized to 100%, corresponding to the LI in the media alone control, and to 0%, corresponding to the LI in the virus alone control. As a functional positive control, recombinant IFN-γ was added at 1.3 IU, 13 IU and 133 IU to virus-containing wells to confirm whether a linear viral suppression dose response could be observed (see Figure 2). All wells were analyzed in triplicate.

### 2.7. Flow Cytometry Analysis

PBMCs from individual volunteer blood samples were stained using the following antibody panel: CD3-PE (BioLegend, San Diego, CA, USA, cat #300441), CD4-PerCP-Cy5.5 (BD Biosciences, Franklin Lakes, NJ, USA, cat#560650), CD8-APC (BD Biosciences cat#566852), CD45RA-Pacific Blue (BioLegend cat# 304123) and CD45RO-PE-Cy7 (BD Biosciences cat#560608). Samples were acquired using BD FACSLyric^TM^ flow cytometer with BD FACSuite^TM^ software (BD Biosciences). FACS files were analyzed using FlowJo software (BD Biosciences, Franklin Lakes, NJ, USA).

## 3. Results

### 3.1. IFN-γ+ Th1 Cell Titers

**Figure 3:**
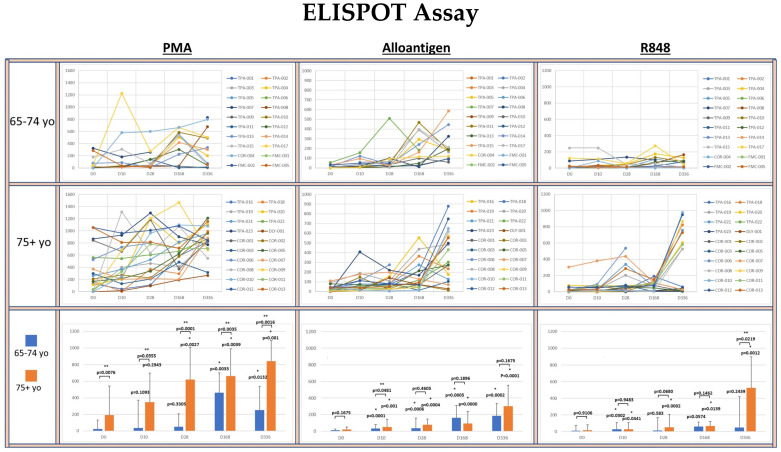
Spider plots indicate the IFN-γ ELISPOT assay results from PBMCs from each volunteer activated with PMA, alloantigen or R848 at baseline, day 10, day 28, day 168 and day 336 segregated by age group. Spots per 0.1 × 10^6^ PBMC are shown. The bar graphs summarize the mean ± SE of the spots from each age group. Significance was analyzed between the age groups at each time point and within each age group at each time point compared to baseline. Statistical analysis was conducted using Student’s *t*-test. *p* values are shown. Significance *p* < 0.05. (*) significantly different than baseline. (**) significant difference between groups.

To investigate whether allo-priming could increase the titer of circulating IFN-γ+ Th1 cells, PBMCs isolated from clinical blood samples were analyzed for their ability to produce IFN-γ after activation using an ELISPOT assay (see Figure 3).

The potential for circulating T cells to produce IFN-γ was determined by polyclonal activation of the PBMCs with PMA. The median number of PMA-activated T cells producing IFN-γ was significantly greater in the 75+ yo group compared to the 65–74 yo group at each time point. Within each age group, the median IFN-γ release was significantly increased at each time point compared to baseline in the 75+ yo group. In the 65–74 yo group, the median potential for IFN-γ also increased at each time point, but it only became significantly greater than baseline at day 168 and day 336.

The number of allo-specific Th1 cells in circulation resulting from the rejection of AlloStim^®^ was determined by activation of PBMCs with a lysate derived from the AlloStim^®^ cells. The amount of Allo-specific Th1 cells was negligible in both age groups at baseline. After allo-prime dosing, the median number of allo-specific Th1 cells significantly increased in both age groups compared to baseline at each time point. The increase in allo-specific Th1 cells in the 75+ yo group was significantly greater than in the 65–74 yo group only at day 10, while at all other time points, the median number of allo-specific cells was not significantly different between the age groups.

To determine the capacity of monocytes in the PBMCs to non-specifically activate bystander T cells after TLR 7/8 activation (simulating an ssRNA viral infection), PBMCs were activated with R848. There was negligible bystander activation of IFN-γ+ cells after R848 activation in both age groups at baseline. There was no significant increase detected in the median number of IFN-γ producing cells after R848 activation compared to baseline in the 65–74 yo group at any time point, except for day 10. In the 75+ yo group, the median number of IFN-γ producing cells after R848 activation was significantly increased compared to baseline at each time point.

### 3.2. Viral Lytic Activity Suppression

**Figure 4:**
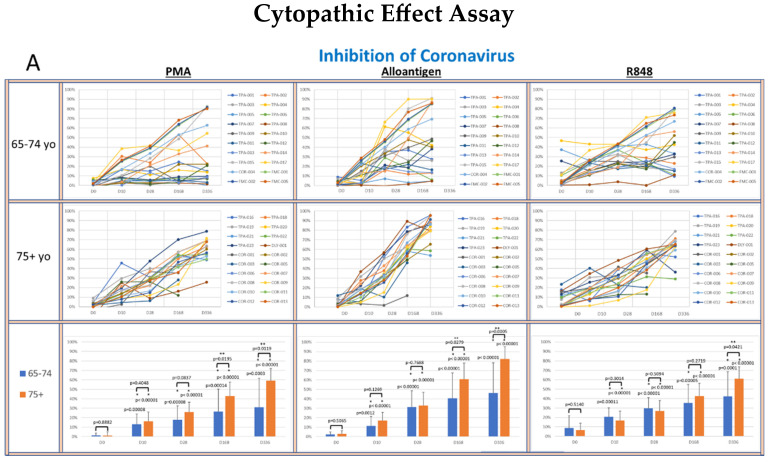

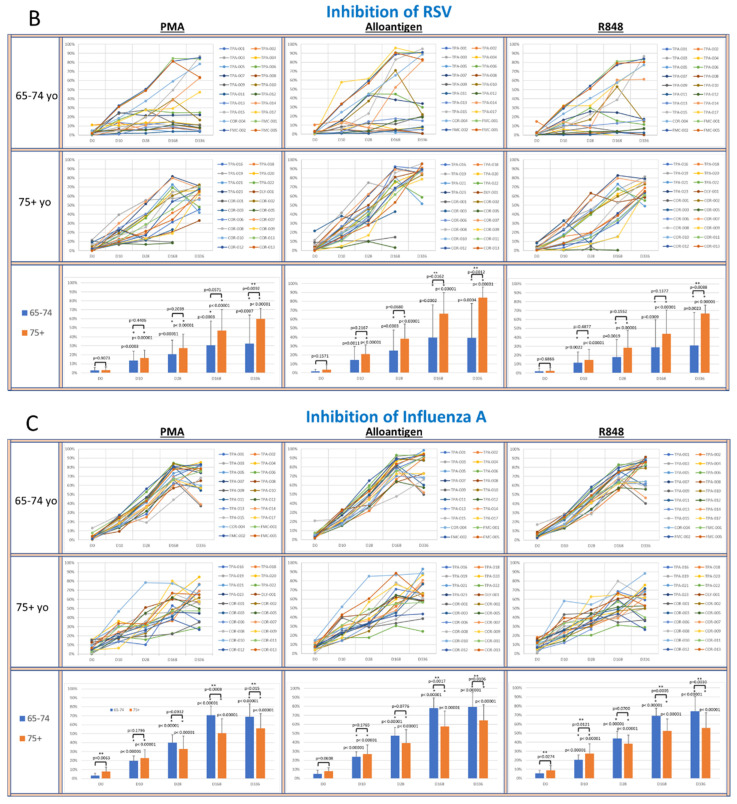

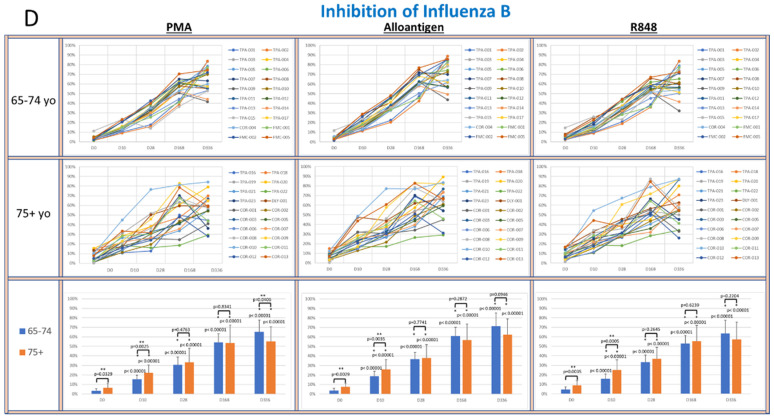
(**A**–**D**): The panels show spider plots of each volunteer indicating the normalized percent viral suppression of supernatants derived from PBMC cultures collected on days 0, 10, 28, 168 and 336 that were activated with PMA, alloantigen and R848 for each age group. The bar charts summarize the mean ± SE of each age group at each time point. The difference between the age groups at each time point and the difference at each time point for each age group compared to baseline were analyzed using Student’s *t*-test. *p* values are shown on the bar charts. *p* values < 0.05 were considered significant. (*) indicates significance compared to baseline. (**) indicates significance between age groups at a single time point.

To determine whether supernatants from activated PBMCs could suppress viral lytic activity in a CPE assay [33], supernatants collected from cultured PBMCs activated with PMA, alloantigen or R848 were co-cultured with live virus cultures. The percent inhibition of viral lytic activity for coronavirus is shown in Figure 4A, RSV in Figure 4B, IAV in Figure 4C and IBV in Figure 4D.

The viral suppressive activity of supernatants from all activation groups toward all tested viruses at baseline was very low (<20%). However, there was significantly better baseline suppression in the 75+ yo age group against IAV and IBV compared to the 65–74 yo group at baseline, with no significant difference at baseline between the age groups for RSV or coronavirus. A consistent self-amplifying pattern of increased viral suppression over time was evident in all the test groups, even after the allo-priming doses were completed.

In the coronavirus CPE cultures (Figure 4A), the viral suppression after PMA and alloantigen became significantly increased in the 75+ yo group compared to the 65–74 yo group on day 168 and day 336 and also significantly increased at day 336 after R848 activation. In the RSV CPE cultures (Figure 4B), the viral suppression in the 75+ yo group compared to the 65–74 yo group was significantly higher at day 336 in all activation groups and significantly increased at day 168 and day 336 after alloantigen activation.

While viral suppression of coronavirus and RSV over time was significantly increased in the 75+ yo group compared to the 65–74 yo group, a different pattern was observed in the influenza cultures. In the IAV CPE cultures (Figure 4C), the viral suppression in the 65–74 yo group compared to the 75+ yo group was significantly higher at day 168 and day 336 in all activation conditions. In the IBV CPE cultures (Figure 4D), the viral suppression was also significantly higher in the 65–74 yo group compared to the 75+ yo group after PMA activation at day 336. At all other earlier time points and activation conditions, viral suppression was amplified over time at statistically equal amounts between the age groups.

### 3.3. Flow Cytometry

The changes in the circulating percentages of CD4+ CD45RO+ and CD8+ CD45RO+ memory T cells were determined via flow cytometry at baseline and days 10, 28, 168 and 336 (see Table 1). No significant change in the proportions of memory cells was found between the age groups at any time point.

### 3.4. SARS-CoV-2 Vaccine IgG Titers

In total, 7 of 19 (37%) evaluable volunteers in the 65–74 yo age group and 4 of 20 (20%) in the 75+ yo age group presented with non-neutralizing SARS-CoV-2 IgG titers at baseline after having been fully vaccinated. Among these low responders, 5 of 7 (71%) in the 65–74 yo group and 1 of 4 (25%) in the 75+ yo group developed IgG neutralizing titers after allo-priming without additional COVID-19 vaccinations.

### 3.5. COVID-19 Incidence

In total, 4 of the 40 (10%) volunteers were found to be positive for COVID-19 antigen at routine office visits during the 336-day observation period after allo-priming, with 3 of these 4 volunteers in the 65–74 yo age group and 1 of the 4 in the 75+ yo age group. The findings were incidental, and none of these COVID-19 antigen positive volunteers reported any symptoms at the time of detection.

### 3.6. Adverse Events

A summary of the reported adverse events (AEs) is shown in Table 2. Overall, 31 of 40 volunteers (77.5%) did not experience any AE during the 336-day observation period. The most frequently reported AE was Grade 1 (mild) rash (10%) and Grade 1 non-COVID flu symptoms (7.5%). One serious adverse event (SAE) was reported in a 74 yo volunteer, who was hospitalized with Grade 2 (moderate) upper respiratory pneumonia due to an unspecified organism (COVID-19, influenza A/B and MRSA were negative; rare Gram-positive bacilli and cocci were present). This volunteer fully recovered and was discharged after 3 days of antibiotic and nebulizer therapy. One volunteer reported Grade 1 anemia. No other clinically significant changes in blood chemistries were reported.

## 4. Discussion

Vaccines generally contain an antigen and an adjuvant. In allo-priming, the alloantigens expressed on the intentionally mis-matched, living Th1 immune cells (AlloStim^®^) serve as the source of antigens, and the activated AlloStim^®^ cells’ expression of high-density surface CD40L and secretion of inflammatory cytokines such as IFN-γ serve as the key adjuvants to steer the allo-specific immune response to a Th1 bias upon rejection by the host [34,35]. The elderly generally present with an imbalance in circulating Th1/Th2 cell ratios, with loss of Th1 cells and IFN-γ [36,37]. The intradermal (ID) injection route was selected based upon prior studies that had shown that viral antigens injected ID tend to drive Th1-biased immune responses to the injected antigens [38].

Loss of the innate cellular immune function in the elderly is associated with a decline in Th1 cells [39]. Th1 cells are a major source of IFN-γ, and a low amount of IFN-γ in response to respiratory viral infection is correlated with the age-related decline in vaccine efficacy and with increases in morbidity and mortality [40]. The ELISPOT assay (see Figure 3) demonstrated increased IFN-γ+ cells in the 75+ yo age group compared to the 65–74 yo age group at baseline, which is inconsistent with the known age-related decline in Th1 cells. This result was likely due to the standardization of cell numbers to be the same in each ELISPOT well for all activation methods. The strong polyclonal PMA activation signal resulted in larger spots, representing clusters of multiple cells, in the younger group compared to the older group. This resulted in an under-reporting of the number of IFN-γ positive spots in the younger group by the image analysis system. This finding was confirmed by review of the spot sizes from the image analyzer and by correlation with the amount of IFN-γ secreted in the supernatant analyzed by ELISA, both being higher in the younger group.

In order to increase the titer of Th1 cells, repeated ID injections of AlloStim^®^ cells were administered. The number of cells capable of releasing IFN-γ in response to an alloantigen pulse was analyzed by the ELISPOT assay. Cells that produced IFN-γ after an alloantigen pulse were significantly increased after allo-priming and continued to increase over time after completion of dosing on Day 14, providing support for the putative primary mechanism of action of allo-priming to enhance Th1 cell titers (see Figure 1).

While the allo-specific Th1 cell titers were significantly increased over time, FACS analyses of T-cell subpopulations did not show any major shifts in CD4+ or CD8+ CD45RO+ memory cells over the same timeframe (see Table 1). This finding is consistent with previous studies demonstrating long-lasting IFN-γ increase over time after bacillus Calmette–Guérin (BCG) vaccination, where heterologous immune (HI) effects were observed without any change in the percentages of T-cell subsets in circulation [41,42]. It is possible that a separate pool of activated memory cells that produce IFN-γ might have migrated to T-cell zones of peripheral lymph nodes or extravasated into tissues, resulting in an underestimation of the CD45RO+ memory cell fractions in the peripheral blood, both in our study as well as in prior studies with BCG vaccination.

The self-amplifying pattern of allo-specific Th1 cells without additional vaccinations suggests that these cells expanded over time in vivo. Since Th1 memory cells can be activated without T-cell receptor (TCR) engagement through a bystander mechanism [43], the significant increases in allo-specific Th1 cells observed are believed to most likely be the result of Th1 cell expansion after bystander activation by cytokines released upon environmental encounters with respiratory viruses. Bystander T-cell activation has been observed during viral-specific immune responses and occurs preferentially among CD4+ memory T cells, such as Th1 cells [43].

Evidence that the bystander activation mechanism of T cells was intact and could be occurring in vivo was demonstrated by the PBMC responses to R848 (Resiquimod) (see Figure 3 and Figure 4). R848 is a TLR 7/8 agonist. Engagement of TLR7/8 receptors in macrophages contained in PBMC cultures mimics their detection of single-stranded RNA (ssRNA) viral infections (e.g., coronaviruses, influenza viruses and RSV). TLR 7/8 signaling in macrophages can induce the production of inflammatory cytokines, such as IL-12 and IL-18 [44], which are capable of non-specifically activating bystander memory T cells, such as memory Th1 cells.

In an in vivo setting, these monokines can potentiate Th1 immune responses to an ongoing infection, including in the elderly [45,46]. The R848-activated T cells analyzed via ELISPOT (Figure 3) and the viral suppression of supernatants from R848-activated cells in the CPE assay (Figure 4) support the notion that TLR7/8 activation of monocytes after ssRNA virus infection could occur in the elderly and result in the release of inflammatory cytokines that subsequently activate bystander Th1 memory cells to release IFN-γ. Bystander IFN-γ release can have a feed-forward effect to activate and expand additional bystander Th1 cells, creating an inflammatory microenvironment [47], which could replicate the early innate immune response that occurs in young individuals in response to respiratory viral infections.

The response pattern of IFN-γ release after R848 activation on the ELISPOT assay showed low expression in the 65–74 yo group compared to the 75+ yo group, which was inconsistent with the response patterns after activation with PMA and alloantigen in this age group. The reason for the apparent poor responsiveness in the younger age group after R848 activation is not clear; however, it may be due to differential numbers of CD56+ NK cells in the PBMC samples. Previous studies have shown that R848 induces IFN-γ expression through activation of NK cells and not T cells [48]. However, if this were the case, it would be expected that the younger group would be more responsive than the older group, as there is an age-related decline in NK cells [49].

The most likely explanation for this unexpected finding is that the timing of the cell harvest may have affected the ELISPOT readout. The PBMCs were harvested 120 h after R848 activation and transferred to an ELISPOT plate for analysis. This timing was optimized for the 75+ yo group, as it was expected that the elderly would be less responsive and may take more time for the bystander activation of T cells to be detected. However, T cells in the younger group may have been activated earlier and stopped producing cytokines at the 120 h time point. This hypothesis is supported by the fact that the supernatants from the 65–74 yo group suppressed viral lytic activity in the CPE assay (Figure 4) with the same self-amplifying pattern as the 75+ yo age group, suggesting that IFN-γ was produced into the supernatant over the 120 h incubation period.

Encounters with environmental pathogens can leave imprints on an immune system modulated by allo-priming, which could then affect future immune responses to different viruses through a trained immunity mechanism [50]. The modulated elderly immune system with restored cellular immune function could be remodeled through a similar mechanism, as has been suggested by the “hygiene hypothesis” [51], which proposes that the building of a healthy immune system occurs through environmental exposures to pathogens [52].

The pattern of viral suppression in the CPE assays was shown to be significantly increased in the 65–74 yo group compared to the 75+ yo group against IAV and IBV, while suppression was significantly increased in the 75+ yo group compared to the 65–74 yo group against RSV and coronavirus. These differences in anti-viral activity observed between the age groups against different viruses could be related to resident trained immunity histories. Different trained immune histories might have resulted in the differential release of anti-viral cytokines upon PBMC activation at the different time points. For example, a trained immunity effect might be more evident in the 65–74 yo group against IAV/IBV due to better responsiveness to influenza vaccination in this group as compared to the 75+ yo group [53,54].

Memory Th1 cells resulting from a primary viral infection can have a beneficial effect on subsequent infection with an unrelated virus through HI mechanisms [55]. Training of innate immune cells, such as monocytes, macrophages and/or natural killer (NK) cells, via infection or vaccination can enhance the immune responses against a new pathogen. For example, trained immunity of alveolar macrophages after viral infection in the lungs is known to support HI responses to unrelated viruses [56].

The self-amplifying increases in cellular immune function and anti-viral protection found here to occur over time, without additional vaccinating doses, support the hypothesis that allo-priming could potentially provide broad respiratory viral protection to the elderly through HI and trained immunity mechanisms. Broad protection against respiratory viruses can occur via HI responses against unrelated subsequent viral infections, including against viruses such as RSV, rhinovirus, coronavirus and influenza viruses [57].

HI broad protection against respiratory viral infections after allo-priming may occur through a similar mechanism, as has been reported after vaccination with BCG. Vaccination with the live BCG vaccine has been shown to provide HI protection against non-related infections [58] and reduce the incidence of respiratory infections in the elderly [59]. HI protection after BCG vaccination occurs through a trained immunity mechanism that results in increased release of IFN-γ upon viral encounter [60]. AlloStim^®^ is a live vaccine that, like BCG, could lead to the long-term presence of memory Th1 cells and increased release of IFN-γ upon viral infection. The increases in Th1 cells observed after allo-priming may thus similarly be associated with HI and trained immunity responses to viral infection, as observed after BCG vaccination [41].

Clinical studies suggest that trained immunity can be utilized to enhance immune responses against infections and improve the efficiency of vaccinations in the elderly [61]. BCG vaccination, for example, has been shown to increase the titers of IgG [62]. In this study, volunteers who presented with non-neutralizing SARS-CoV-2 IgG titers at baseline, after having been fully vaccinated against the virus, developed IgG neutralizing titers after allo-priming without additional COVID-19 vaccinations. T-cell responses are positively correlated with the levels of neutralizing antibodies after vaccination [63]. The increased cellular immune function demonstrated in the ELISPOT and CPE assays in this study (see Figure 3 and Figure 4) and the increase in COVID-19 IgG titers suggest that allo-priming may also have an effect on vaccine responsiveness in the elderly.

In 2023 when the volunteers in this study were being evaluated, adults aged 65 and older in the United States were significantly impacted by COVID-19. This elderly age group accounted for approximately 62.9% of COVID-19 hospitalizations, 61.3% of intensive care unit admissions and 87.9% of in-hospital deaths [64]. During the 336-day observation period in this study, 10% of the volunteers tested positive for COVID-19 antigen on routine clinic visits without exhibiting any symptoms. An additional two volunteers presented to the clinic with mild non-COVID-19 flu-like symptoms. One volunteer was hospitalized for respiratory symptoms over the observation period and fully recovered. These real-life observations support the hypothesis that the enhanced cellular immune function that occurs after allo-priming may provide broad anti-viral protection against serious disease from respiratory viral infection in this population.

The presumptive HI mechanism of allo-priming would have the advantage of potentially reducing the need for multivalent vaccines or advanced knowledge of the antigenic structure of circulating viruses in order to control community-acquired viral infections. A vaccine with a HI mechanism would also potentially eliminate the need for frequent re-formulations of some vaccines in order to maintain protection against viral mutant strains and could also possibly provide protection from a future, currently unknown, pandemic viral pathogen (“Disease X”) [65].

## 5. Conclusions

This phase I/II clinical trial provided a clinical proof of concept for the use of allo-priming as a strategy to possibly protect the vulnerable elderly population from severe symptoms resulting from respiratory viral infection. The proof-of-concept data, together with the excellent safety profile, allow the consideration for future clinical testing to evaluate the effects of allo-priming in high-risk populations, such as assisted living facilities for the elderly, in order to determine, in a randomized controlled study, whether allo-priming will prevent hospitalization and serious disease from community-acquired viral infections.

## Figures and Tables

**Table 1:** The mean ± SE percentage of CD4+ and CD8+ T cells with CD45RO memory phenotype are shown for each age group at each sample day. Changes from baseline and between age groups at each time point were evaluated using Student’s *t*-test. No comparisons met the significance threshold of *p* < 0.05.

Memory Cells						
		D0	D10	D28	D168	D336
65–74 yo (*n* = 20)	%CD4+ CD45RO+	65.3 ± 15.5	63.0 ± 13.2	69.3 ± 12.0	56.7 ± 8.3	57.8 ± 13.5
	%CD8+ CD45RO+	39.2 ± 20.8	34.5 ± 14.8	40.1 ± 19.1	33.3 ± 12.6	36.7 ± 18.0
75+ yo (*n* = 20)	%CD4+ CD45RO+	62.9 ± 14.9	66.9 ± 15.7	69.0 ± 13.6	69.3 ± 16.6	63.5 ± 17.3
	%CD8+ CD45RO+	41.3 ± 16.9	43.8 ± 16.1	43.1 ± 20.5	43.8 ± 18.9	34.5 ± 2.6

**Table 2:** The most frequently adverse events reported by investigators during the 336-day follow-up period are listed by incidence and percent of total (*n* = 40) and graded in accordance with CTCAE v.5.

Adverse Events		
Event	Grade	#Subjects (Percent)
Rash (all)	1	4 (10%)
Non-COVID Flu Symptoms	1	3 (7.5%)
Back Pain	1	2 (5%)
Anemia	1	2 (5%)
Urinary Tract Infection	1	1 (2.5%)
Vasovagal Syndrome	1	1 (2.5%)
Epistaxis	1	1 (2.5%)

## Data Availability

The raw data supporting the conclusions of this article will be made available by the authors on request.

## Abbreviations

The following abbreviations are used in this manuscript:

ATCC	American Type Culture Collection
Cat#	Catalog Number
CTCAE	Common Terminology Criteria for Adverse Events
CTL	Cytotoxic T-Lymphocyte
EDTA	Ethylenediaminetetraacetic Acid
EIA	Enzyme Immunoassay
FACS	Fluorescence-Activated Cell Sorting
Th1	T Helper 1 Lymphocyte
Th2	T Helper 2 Lymphocyte
TLR	Toll-Like Receptor
yo	Years Old
